# Increased behavioral problems in children with sleep-disordered breathing

**DOI:** 10.1186/s13052-022-01364-w

**Published:** 2022-09-15

**Authors:** Eszter Csábi, Veronika Gaál, Emese Hallgató, Rebeka Anna Schulcz, Gábor Katona, Pálma Benedek

**Affiliations:** 1 Department of Cognitive and Neuropsychology, Institute of Psychology, University of Szeged, Szeged, Hungary; 2 Department of Pediatrics, Clinical Center, University of Pécs, Pécs, Hungary; 3 Sleep Disorders Laboratory of Heim Pal National Pediatric Institute, Budapest, Hungary; 4 Department of Oto-Rhino- Laryngology and Head- Neck Surgery, University of Szeged, Szeged, Hungary

**Keywords:** Sleep-disordered breathing, Behavior dysregulation, Hyperactivity, Inattentiveness children, Snoring, Sleep apnea

## Abstract

**Background:**

Healthy sleep is essential for the cognitive, behavioral and emotional development of children. Therefore, this study aimed to assess the behavioral consequences of sleep disturbances by examining children with sleep-disordered breathing compared with control participants.

**Methods:**

Seventy-eight children with SDB (average age: 6.7 years (SD = 1.83); 61 had OSA and 17 had primary snoring) and 156 control subjects (average age: 6.57 years (SD = 1.46) participated in the study. We matched the groups in age (*t*(232) = 0.578, *p* = 0.564) and gender (*χ*^*2*^(1) = 2.192, *p* = 0.139). In the SDB group, the average Apnea–Hypopnea Index was 3.44 event/h (SD = 4.00), the average desaturation level was 87.37% (SD = 6.91). Parent-report rating scales were used to measure the children’s daytime behavior including Attention Deficit Hyperactivity Disorder Rating Scale, Strengths and Difficulties Questionnaire, and Child Behavior Checklist.

**Results:**

Our results showed that children with SDB exhibited a higher level of inattentiveness and hyperactive behavior. Furthermore, the SDB group demonstrated more internalizing (anxiety, depression, somatic complaints, social problems) (*p* < 0.001) and externalizing (aggressive and rule-breaking behavior) problems compared with children without SDB, irrespective of severity.

**Conclusions:**

Based on our findings we supposed that snoring and mild OSA had a risk for developing behavioral and emotional dysfunctions as much as moderate-severe OSA. Therefore, clinical research and practice need to focus more on the accurate assessment and treatment of sleep disturbances in childhood, particularly primary snoring, and mild obstructive sleep apnea.

## Background

Sleep-disordered breathing (SDB) is characterized by a broad spectrum of pathology, ranging from partial upper airway obstruction such as primary snoring, to complete upper airway obstruction such as obstructive sleep apnea (OSA). Primary snoring is at the mildest end of the spectrum and is defined as habitual snoring without respiratory events, abnormalities of gas exchange, or evidence of sleep disruption [1]. OSA is described as prolonged and intermittent upper airway obstruction during sleep resulting in hypoxia, hypercapnia and fragmented sleep [1]. The prevalence of snoring in children ranges from 5 to 12%, of which 5% have OSA [2]. Moreover, Castronovo et al. [3] found that the prevalence of SDB is up to 34.5% [3]. Several medical conditions contribute to the development of SDB such as adenotonsillar hypertrophy, obesity, craniofacial malformation (e.g., Crouzon syndrome), neuromuscular disorders (e.g., muscle hypotonia) or any anatomical abnormalities that narrow the upper airway (e.g., tongue base hypertrophy) [4]. There is emerging evidence that SDB is associated with a deficit in cognitive functioning such as attention [5], information processing [6], executive functions [7–9] and memory [10–12]. These decrements can lead to decreased learning abilities, lower general intelligence [13] and poor academic performance which are frequently observed in children with SDB [14, 15].

Sleep fragmentation and oxygen deprivation might cause cognitive dysfunction by disturbing neuronal myelination and normal blood gas which impacts the development of the brain, particularly in the hippocampus and frontal lobe structures [7, 16, 17]. Previous studies found that the frontal lobe is uniquely vulnerable to the pathological effect of SDB [7, 16]. The frontal lobe develops throughout childhood and is crucial for executive functions which are responsible for higher-order human functioning, self-regulation, inhibition or social interaction. Thus, damage to this region during the maturation period could affect behavioral functioning [18, 19].

The problem with behavioral regulation exhibited by children with SDB also implies frontal lobe dysfunction. Cross-sectional studies have found that children with SDB frequently demonstrate inattention, hyperactivity [20, 21], externalizing problems such as aggression or rule-breaking behavior [6, 22–24] and internalizing problems including anxiety, depression, somatic complaints [5, 21, 24] or social problems [5]. Previous studies suggest that behavioral problems are irrespective of severity [23, 24].

Recent studies supposed that sleepiness might also affect the regulation of emotions and thus could result in impulsivity, irritability and hyperactive behavior [4, 25]. Chervin et al. [25] revealed that snoring and excessive daytime sleepiness related to increased inattention and hyperactive behavior could likely lead to attention-deficit/hyperactivity disorder (ADHD). A meta-analysis by Sedky et al. [20] demonstrated that children with SDB are at a higher risk of presenting ADHD symptoms, such as inattention and hyperactivity. From another perspective, children with ADHD appear to exhibit more sleep difficulties (e.g., delayed sleep onset, bedtime resistance, tiredness during wakefulness) and symptoms of SDB [26].

The current study aims to investigate the behavioral consequences of SDB compared with control participants without SDB. We hypothesized that children with SDB exhibited more behavioral problems than the age-matched control group regardless of the severity of SDB.

## Methods

### Participants

Seventy-eight children with SDB participated in the SDB group. They were recruited from amongst patients who visited the sleep laboratory of the hospitals. All of them were untreated before the study. Children with a history of developmental, psychiatric or neurological disorders or who were on medication known to affect sleep were excluded. After the physical examination, all of them underwent overnight polygraphy, which was performed using the Somnomedics SOMNOscreen plus device (Randersacker, Germany) at the Sleep Disorders Laboratory of Heim Pal National Pediatric Institute, Budapest, Hungary, and at the Department of Pediatrics, Clinical Center, University of Pécs, Hungary. SDB was diagnosed by a board-certified sleep physician. Patients who met the International Classification of Sleep Disorders criteria [1] for SDB were included in the study. Furthermore, we used the OSA18 Questionnaire to measure the quality of life and to identify the daytime and nighttime symptoms of SDB [27]. The average age of the SDB group was 6.7 years (SD) = 1.83) (minimum 4 to maximum 10 years; 32 females/46 males), of which 61 had OSA and 17 had primary snoring. The average Apnea–Hypopnea Index was 3.44 event/h (SD = 4.00) (minimum 0 to maximum 19.3 event/h), the average desaturation level was 87.37% (SD = 6.91) (minimum 60% to maximum 99%), and the average body mass index was 17.13 kg/m^2^ (SD = 6.78). According to previous studies, primary snoring has been linked to daytime functional and behavioral impairments [5, 14, 25]. Therefore, we compared the performance of the SDB group to that of the controls and did not examine the OSA and snoring subgroups separately.

The control group consisted of 156 children (average age: 6.57 years (SD = 1.46), 80 females/76 males). The control group was recruited through collaboration with public schools. We matched the two groups on age (*t*(232) = 0.578, *p* = 0.564) and gender (*χ*^*2*^(1) = 2.192, *p* = 0.139). They did not suffer from any developmental, psychiatric, or neurological disorders and were free of any sleeping disorders.

Based on the OSA18 questionnaire [27] we excluded those children from the control group who reported SDB-like symptoms and reached at least 60 points or more on the questionnaire. The comparison of the SDB and control groups is presented in Table 1.

**Table 1:** Mean Ranks and medians of OSA-18 subscales

	SDB group^a^		Control group^b^		*P*
	Mean Ranks	Median	Mean Ranks	Median	
Sleep Disorders	195.5	15	78,5	4	< .001
Physical Distress	175.56	15	88.47	7	< .001
Diurnal Problems	163.31	8,5	94.59	4	< .001
Emotional Distress	165.86	10	93.32	4	< .001
Caretaker Preoccupation	181.47	13,5	85.51	4	< .001
Total score of OSA-18	192.8	72	79.85	34	< .001

^a^*N* = 78, ^b^*N* = 156

### Behavioral assessment

#### Attention Deficit Hyperactivity Disorder Rating Scale (ADHD-RS)

ADHD-RS is an 18-item rating scale designed to assess symptoms of inattention, impulsivity and hyperactivity disorder. Each item is rated on a 4-point scale. The rating scale consists of two subscales based on the relevant symptoms: Inattention problems and Hyperactivity-Impulsivity [28]. We used the parent rating scale version in the study.

#### Strengths and Difficulties Questionnaire (SDQ)

SDQ is a 25-item rating scale used to measure emotional and behavioral problems. Each item is rated on a 3-point scale. The SDQ has five subscales, four of them measure major difficulties commonly experienced by children (Hyperactivity, Emotional Symptoms, Behavioral Problems, Social Problems). One subscale assesses strengths (Prosocial Behavior). There are three versions of the scale: parent-report, teacher-report and self-report versions. This study was based on the parent-report version of the scale [29].

#### Child Behavior Checklist (CBCL)

CBCL is designed to assess behavioral and emotional problems and competencies. The Hungarian version of the questionnaire consists of 46 items, each item is rated on a 3-point scale. The CBCL measures two factors: Internalizing, composed of subscales Anxious/Depressed, Somatic Complaints, Inattention Problems and Social Problems; and Externalizing, consisting of Aggressive Behavior, Deviant/Rule-Breaking Behavior. The CBCL is available in three versions: parent report, teacher report, and self-report versions. The parent version of the scale was used [30].

#### OSA18 questionnaire

OSA-18 is a caregiver-administrated quality of life survey that contains 18 items [27]. The questionnaire is organized into five domains: sleep disorders, physical distress, emotional distress, diurnal problems and caretaker preoccupation. Each domain can be scored on a 7-point Likert scale. The OSA-18 total score is the sum of all 18 items. According to Franco et al. [27], a total score < 60, between 60 and 80, and above 80 suggests a small impact on the quality of life, moderate impact and large impact, respectively.

### Polysomnographic measurement

The study was performed using the Somnomedics SOMNOscreen plus device (Somnomedics) according to the guideline. Polygraphic readings were evaluated by a physician experienced in sleep medicine. Breathing irregularities (apneas and hypopneas) were analyzed and Apnea–Hypopnea Index (AHI) was presented as the number of apneas and hypopneas per sleeping hour. The desaturation index indicates the number of periods with desaturation (minimum of 3% fall in oxygen saturation) per hour. According to the polysomnographic diagnostic criteria for childhood OSA by Marcus et al. [1] one episode of apnea during sleep is considered pathologic. The respiratory pause has to last for at least two breaths to be considered abnormal. In hypopnea, the respiratory amplitude is reduced by at least 30% and occurs when the oxygen saturation drops by 3%. We classified the severity of OSAS as mild, AHI = 1 event/hour with desaturation or AHI 2–5 event/hour; moderate, 5 < AHI > 10 event/hour; and severe AHI > 10 event/hour [31].

### Procedure

All children with SDB underwent a standard pediatric overnight clinical polygraphy. Medical histories and physical examinations were conducted by a board-certified sleep physician before the diagnostic night. On the night of the study, the parents, most often the mothers, completed the demographic and behavioral questionnaires. Written informed consent was obtained from both parents, and procedures were verbally explained to the children before the commencement of the study. The participants did not receive any financial compensation for their participation. Ethics approval was obtained by the Ethics Committee at Heim Pal National Pediatric Institute, Budapest (number of the Ethical Approval: KUT-6/2017).

### Statistical analysis

Statistical analysis was performed using the SPSS 22.0 (Statistical Package for the Social Sciences). Gender proportions were compared between the groups using Pearson’s chi-square test. Group differences were tested with the Mann–Whitney U test. Spearman’s correlation was used to explore the relationship between respiratory variables and behavioral measurements.

## Results

The figure represents medians: a) ADHD-RS Rating Scale: Attentional Problem (AP), Hyperactivity-Impulsivity (HI). b) Strength and Difficulty Questionnaire (SDQ): Hyperactivity (H), Emotional Problems (ES), Behaviour Problem (BP), Social Problem (SP), Prosocial Behaviour (PB). c) Total Score of SDQ (TSS). d) Child Behavior Checklist (CBCL): Social Problems (SP), Anxious/Depressed (AD), Rule-Breaking Behavior (RBB), Aggressive Behaviour (AB), Somatic Complaints (SC), Attentional Problems (AP). e) Total Score of CBCL. *p* < 0.001***, *p* < 0.05**.

### ADHD rating scale

**Fig. 1:**
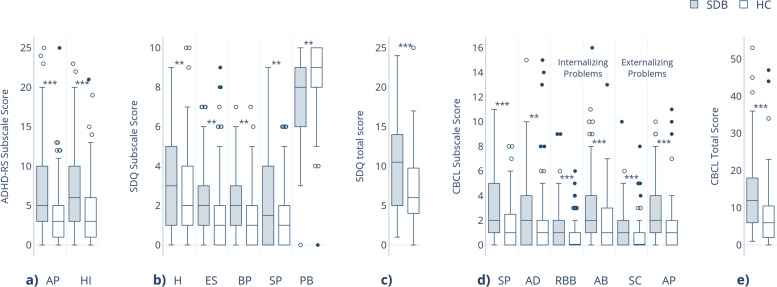
Comparison of the behavioural assessment between SDB and healthy control (HC) groups

The SDB group demonstrated significantly higher ratings than the control group on both subscales of ADHD-RS: Attentional problems (*U* = 3833, *z* =-4.638, *p* < 0.001) and Hyperactivity-Impulsivity (*U* = 3899, *z* = -4.498, *p* < 0.001). The medians are presented in Fig. 1.

### Strength and Difficulty Questionnaire

We revealed a significant effect of group on all SDQ subscales. The SDB group demonstrated significantly higher rates on Hyperactivity (*U* = 4631, *z* = -2.943, *p* = 0.003), Emotional Symptoms (*U* = 4548, *z* =-3.234, *p* = 0.001), Behavior problems (*U* = 4437, *z* = -3.234, *p* = 0.001) and Social problems (*U* = 4716, *z* = -2.917, *p* = 0.004). Furthermore, children with SDB showed significantly lower rates on the subscales of prosocial behavior than the control participants (*U* = 4589, *z* = -3.136, *p* = 0.002). The Total number of SDQ was significantly higher in the SDB group than in the control group (*U* = 3852, *z* = -4.526, *p* < 0.001) (for medians see Fig. 1).

### Child Behavior Checklist

The subscales of externalizing (*U* = 4041.500, *z* = -4.241, *p* < 0.001) and internalizing problems (*U* = 3893, *z* = -4.522, *p* < 0.001) significantly differed between the two groups. The SDB group demonstrated significantly higher scores than the control group on the subscales of Social Problems (*U* = 3954.500, *z* = -4.449, *p* < 0.001), Anxious/Depressed (*U* = 4496, *z* = -3.373, *p* = 0.001), Aggressive Behavior (*U* = 4149, *z* = -4.046, *p* < 0.001) and Rule-Breaking Behavior (*U* = 4480, *z* = -3.681, *p* < 0.001). Furthermore, the SDB group had significantly higher rates on Somatic Complaints (*U* = 4524, *z* = -3.723, *p* < 0.001) and Attentional Problems (*U* = 3978.500, *z* = -4.443, *p* < 0.001). The Total Problems Summary Scale was higher in the SDB group compared to the control group (U = 3516, z = -5.269, p < 0.001). The medians are presented in Fig. 1.

## Discussion

Our goal was to examine the behavioral consequences of SDB compared to control participants without SDB. Our results revealed that children with all severities of SDB demonstrated a significantly higher level of inattention, hyperactivity-impulsivity, internalizing (social problems, anxiety, depression, somatic complaints) and externalizing problems (rule-breaking behavior, aggression) than control subjects. Consistent with these results, children with SDB exhibited significantly less prosocial behavior.

Our results are in line with earlier studies showing similar irregular behavioral performance in children with SDB compared to controls, [20–22] irrespective of severity [23, 24, 32]. A study by Rosen et al. [24] also found that increased behavioral morbidity was similar between children with primary snoring and children with OSA. Both groups showed significantly more behavioral problems than the control subjects, such as hyperactivity, emotional lability, somatic complaints, oppositional, aggressive behavior and social problems. The authors revealed that externalizing acting out behavior such as hyperactive, oppositional and aggressive behavior showed the most robust association with SDB [24]. In contrast, some studies supposed that the duration of the disease and severity may contribute to the development of more serious behavioral outcomes [22, 33]. Mulvaney et al. [22] provided evidence that an increased frequency of aggressive behavior, inattention and social problems are related to high Respiratory Disturbance Index (RDI—occurrence of apneas and hypopneas).

Contrary to these findings, Jackman et al. [34] revealed that primary snoring and mild OSA are associated with a higher level of internalizing problems than moderate or severe types of OSA. The authors supposed that the parents of the children diagnosed with mild SDB had more unrelated psychological distress than the parents of children who showed more severe SDB symptoms, which engendered more behavioral problems in the children themselves or caused parents to describe their children's behavior in a more negative light [27]. In line with these results, Lewin et al. [6] demonstrated that children with mild OSA had more symptoms of anxiety, depression, social and externalizing problems than children with severe OSA. The authors suggest that their results might be explained by the fact that the severe OSA group did not show abnormalities in the organization of sleep stages; however, they had significantly more arousals than the mild OSA group [6].

A recent study by Smith et al. [35] suggests that frequent snoring is a more effective predictor of behavioral outcomes than AHI. The authors found that behavioral problems increased with the frequency of snoring up to occasional snoring (at least two nights per week) but did not increase based on AHI severity. These results emphasized the importance of examining snoring in the assessment of potential behavioral outcomes in SDB. To summarize, there has been inconsistency across studies in the relationship between the severity of SDB and behavioral consequences. Therefore, future studies need to clarify the effect of the severity of SDB on behavioral functioning and the role of individual vulnerability.

The mechanisms causing the behavioral consequences have not been fully delineated. Some investigators suggest that hypoxia impacts the development of the central nervous system, particularly the hippocampus and frontal lobe structures [7, 16]. Others postulated that sleep fragmentation produces behavioral problems [14]. A few studies assumed that daytime sleepiness is associated with impaired behavior, especially hyperactivity and externalizing acting-out behavior like irritability and impulsivity [4, 25]. However, excessive daytime sleepiness is a less common symptom in children with SDB. In children, sleepiness is frequently acted out behaviorally rather than expressed verbally (e. g. complaining of being tired) [4]. Our findings are consistent with those studies that suggest an overlap between the impairment associated with SDB and the diagnostic criteria for ADHD [20]. However, is still unclear whether sleep disturbances are intrinsic to ADHD or sleep disorders cause ADHD-like symptoms. In the future, we need to clarify whether SDB is intrinsic to ADHD or SDB causes ADHD-like symptoms to avoid misdiagnosis. Therefore, children should be examined for SDB when considering a diagnosis of ADHD.

The main limitation of the study is the lack of overnight polygraphic measurement of the control group. With these data, we would have a deeper insight into the differences and effects of pathological and non-pathological ventilation during sleep on daytime functioning. Nonetheless, our results suggest that mild SDB (e. g., snoring) has a risk of developing behavioral and emotional dysfunctions as much as moderate or severe SDB. Overall, this study highlights that clinical practice need to focus more the adequate treatment of sleep disturbances in childhood, particularly primary snoring, and mild obstructive sleep apnea.

## Conclusion

Our findings revealed that children with SDB demonstrated higher levels of emotional (e g., anxiety, depression, somatic complaints) and behavioral difficulties (e. g., inattentiveness, hyperactive, aggressive and rule-breaking behavior) compared with children without SDB, irrespective of severity. We believe that our study provides evidence that the potential impact of mild SDB may be greater than what was previously believed. Our results confirm the findings of previous studies suggesting that even snoring and a mild level of SDB can increase the risk of behavioral and emotional problems. Our study underlines the importance of early and accurate assessment and adequate treatment of primary snoring as much as moderate or severe type of SDB.

## Data Availability

The full data set and other materials related to about this study can be obtained from the corresponding author on reasonable request.

## Abbreviations

SD: Standard Deviation
SDB: Sleep-Disordered Breathing
OSA: Obstructive Sleep Apnea
ADHD: Attention Deficit and Hyperactivity Disorder
ADHD-RS: Attention Deficit Hyperactivity Disorder Rating Scale
SDQ: Strengths and Difficulties Questionnaire
CBCL: Child Behavior Checklist
AHI: Apnea–Hypopnea Index
RDI: Respiratory Disturbance Index
